# Essential Work of Fracture and Evaluation of the Interfacial Adhesion of Plasticized PLA/PBSA Blends with the Addition of Wheat Bran By-Product

**DOI:** 10.3390/polym14030615

**Published:** 2022-02-04

**Authors:** Laura Aliotta, Alessandro Vannozzi, Patrizia Cinelli, Maria-Beatrice Coltelli, Andrea Lazzeri

**Affiliations:** 1 Department of Civil and Industrial Engineering, University of Pisa, 56122 Pisa, Italy; alessandrovannozzi91@hotmail.it (A.V.); patrizia.cinelli@unipi.it (P.C.); andrea.lazzeri@unipi.it (A.L.); 2 National Interuniversity Consortium of Materials Science and Technology (INSTM), 50121 Florence, Italy; 3 Planet Bioplastics s.r.l., Via San Giovanni Bosco 23, 56127 Pisa, Italy

**Keywords:** biocomposites, natural fibers, poly(lactic acid) (PLA), fracture mechanics

## Abstract

In this work biocomposites based on plasticized poly(lactic acid) (PLA)–poly(butylene succinate-*co*-adipate) (PBSA) matrix containing wheat bran fiber (a low value by-product of food industry) were investigated. The effect of the bran addition on the mechanical properties is strictly correlated to the fiber-matrix adhesion and several analytical models, based on static and dynamic tests, were applied in order to estimate the interfacial shear strength of the biocomposites. Finally, the essential work of fracture approach was carried out to investigate the effect of the bran addition on composite fracture toughness.

## 1. Introduction

In the last years a growing interest towards the development of new advanced bio-based polymeric products that are sustainable, eco-friendly, eco-efficient and biodegradable has arisen for proposing valid alternatives in the global market, that is at present mainly dominated by petroleum-derived products. Thanks to new governments regulations not only researchers but also industries are seeking for more ecologically and friendly materials [1,2,3]. There are several market branches extremely valuable for biodegradable plastic materials such as single use items and those applications where collection and recycling are difficultly achieved, and then for a correct waste management compostability becomes an advantage; for this reason, it is expected that the biodegradable and biobased polymer market will increase in the coming years [4,5].

In this context, among biobased and biodegradable matrices which are industrially compostable and commercially available on the market, poly(lactic acid) (PLA) is one of the most attractive due to its relatively low production cost compared to other biobased polymers [6,7]. PLA exhibits very interesting mechanical properties (about 3 GPa as Young’s modulus, 60 MPa as tensile strength and an elongation at break between 3–6%) [8,9]; however, it also presents some drawbacks to be overcome to reach end-users demands. In particular, PLA is very stiff and brittle and for this reason the addition of plasticizers and/or rubber particles is often required to enhance its elongation at break and tensile toughness [10,11,12,13].

An economic and assessed method to tune polymeric matrix properties is the polymers blending [14]. In literature many flexible biobased polymers have been successfully blended with PLA such as: poly(butylene succinate) (PBS) [15,16,17], poly(butylene succinate-*co*-adipate) (PBSA) [18,19,20], polycaprolactone (PCL) [21,22,23] and poly(butylene adipate-*co*-terephthalate) (PBAT) [24,25].

In this article, on the basis of a previous study [18], a PLA/PBSA blend containing 60 wt.% of PLA and 40 wt.% of PBSA was selected as matrix due to its good flexibility and fracture properties. To this binary blend a by-product natural filler has been added. It is generally expected that the main address for a composite material is to exhibit enhanced physical and mechanical properties when it is compared to its individual components [26]. Nevertheless, in the case of very short fiber or particulate composites, the fibers cannot bear efficiently the load and their randomly orientation does not allow specific reinforcements along the fibers direction. In this case the benefit is related to the cost savings, lighter product weight, valorization of waste products and degradability promotion (in particular the final product disintegration) [27,28,29,30,31].

In response to the demand for extending biobased polymers applications while reducing the final materials cost, different studies are present in literature where the incorporation of low-cost and highly-available natural fillers and short fibers (derived from agricultural and or industrial waste) into a biobased polymeric matrices has been investigated [32,33,34,35]. More research is ongoing to optimize the valorization of agriculture residues as fillers in bio-composites. At this purpose, due to the large amount of grain by-products generated by the main food production, bran is very interesting due to its low cost and wide availability and it is a relevant cellulosic based filler for bioplastic production [36,37,38,39].

To improve the filler dispersion in the polymeric matrix and at the same time to counterbalance the stiffening effect caused by the bran addition, also improving the processability, a plasticizer was added. In particular, Triacetin was added considering the very interesting results found in literature in which Triacetin was added as plasticizer in natural fiber composites [40,41,42]. Ibrahim et al. [1], for example, demonstrated that PLA biocomposites with good mechanical and thermomechanical properties can be obtained using kenaf bast fiber as reinforcement and Triacetin as plasticizer. Furthermore, Pelegrini et al. [43] observed that Triacetin is a good processing aid and coupling agent that does not influence negatively the PLA biocomposites degradation.

In this study, after a preliminary characterization of wheat bran in which its granulometry and aspect ratio was determined; the effect due to the addition of different bran amount (from 10 to 30 wt.%) in a PLA/PBSA based polymeric matrix (plasticized with a fixed amount of Triacetin) was studied. Analytical models were applied in order to predict the mechanical characteristics and the interface adhesion between fibers and matrix that is fundamental to understand the interactions existing between the bran particles and the surrounding matrix. The mechanical properties, in fact, are connected to the fiber and matrix strengths, to the fibers distribution and to the interfacial shear strength (IFSS or τ) [6,27,44,45]. The last parameter, IFSS, is strictly related to different factors such as the interface thickness, the adhesion strength and the energy surface filler [27,46,47]. An IFSS good evaluation was obtained by the application of analytical models based on static and dynamical mechanical tests. Furthermore, for the first time for this biocomposites typology, the fracture toughness and crack resistance were investigated by the essential work of fracture (EWF) approach [48].

## 2. Theoretical Analysis

Two analytical approaches were followed: one based on the application of analytical models taking the data from the static tensile tests, and one taking the data from dynamical mechanical tests.

### 2.1. Analytical Predictive Model Based on Static Tests

For rigid fillers and for ultra-short fibers composites the effect of the fiber content correlated to the fiber/matrix adhesion on the composite stress at break lies among two limits called upper and lower bound [49]. Where low adhesion exists, the load is sustained only by the polymeric matrix and a simple expression (derived by Nicolais and Nicodemo [50]) can be written (Equation (1)):

(1) $$\sigma_{c}=\sigma_{m}\left(1-1.21\;V_{f}^{\frac{2}{3}}\right)\;$$

In Equation (1) *σ_c_* and *σ_m_* are, respectively, the composite strength and the matrix strength while *V_f_* is the filler volume fraction. This equation represents the lower bound for the prediction of the tensile strength of not well bonded particulate filled composites. On the other hand, the upper bound can be obtained considering that the filler decreases the load bearing capacity of the matrix (Equation (2)) [49,51].

(2) $$\sigma_{c}=\sigma_{m}\left(1-V_{f}\right)\;$$

Pukanszky et al. [52,53] provided an empirical relationship for composites strength prediction (Equation (3)) where an empirical constant, named *B*, was correlated to: the particles surface area, particles density and interfacial bonding energy. However, the *B* parameter does not provide a numerical value of the interfacial shear stress (MPa), it is able to give information about the filler/matrix adhesion. In fact, a value of *B* equal or very close to zero corresponds to poor interfacial bonding where the fillers will not carry any load.

(3) $$\sigma_{c}=\sigma_{m}\frac{1-V_{f}}{1+2.5V_{f}}\exp\left(BV_{f}\right)\;$$

By writing Equation (3) in a linear form (Equation (4)), is obtained a linear correlation where the interaction parameter, *B*, corresponds to the slope of the Pukánszky’s plot (obtained plotting the natural logarithm of Pukánszky’s reduced strength, *σ_red_*, against the volume filler fraction).

(4) $$ln\sigma_{red}=ln\frac{\sigma_{c}\left(1+2.5V_{f}\right)}{\sigma_{m}\left(1-V_{f}\right)}=BV_{f}\;$$

Lazzeri and Phuong [54] correlated the Pukánszky’s interaction B parameter with the interfacial shear stress (*IFFS*). In particular, for composites having a length below the critical length (*L_c_*) the failure of the composite occurs by plastic flow of the matrix and the interfacial shear strength (*IFSS*) can be calculated with the following expression:

(5) $$\tau \;\left(or\;IFSS\right)=\frac{2\sigma_{m}\left(B-2.04\right)}{\eta_{0}a_{r}}\;$$

where *η_0_* is the fiber orientation factor, *a_r_* is the fibers aspect ratio. In the case of particulate fillers the fibers orientation factor and the fibers aspect ratio can be taken equal to one; for very short aspect ratio fibers (such as those used in this work), a randomly fiber orientation can be considered and a value *η*_0_ = 3/8 can be taken [27,55]. It is evident, from Equation (5), that the *IFSS* is directly proportional to *B* and 2.04 is the lower limit for the *IFSS* estimation.

### 2.2. Analytical Predicitve Model Based on Dynamic Tests

The interfacial strength between the polymeric matrix and the different volume content of wheat bran added, can be tracked using the so called “adhesion factor” (*A*). The damping factor (*tanδ*), obtainable from DMTA tests, is an indicator of the material molecular motions. Thanks to its estimation it is possible to have an idea of the fiber-matrix interfacial bonding. The adhesion parameter was introduced by Kubat et al. [56] starting on the assumption that the mechanical loss factor (*tanδ_c_*) of the composite can be given as follows (Equation (6)):

(6) $$tan\delta_{c}=V_{f}tan\delta_{f}+V_{i}tan\delta_{i}+V_{m}tan\delta_{m}\;$$

where the subscripts *f*, *i*, and *m* subscripts denote the filler, the interphase, and matrix; while *V_f_* is the volume filler fraction. Making the assumption that the volume fraction of the interphase is rather small (*δ_f_* ≈ 0), its contribution can be neglected and Equation (6) can be rearranged as follows [57] (Equation (7)):

(7) $$\frac{tan\delta_{c}}{tan\delta_{m}}\approx \left(1-V_{f}\right)\left(1+A\right)\;$$

with the “*A*” term, called adhesion factor. Equation (7) can be rewritten in order to explicit the adhesion factor as follows (Equation (8)):

(8) $$A=\frac{1}{1-V_{f}}\;\frac{tan\delta_{c}}{tan\delta_{m}}-1\;$$

Calculating the adhesion factor from DMTA experiments, it is possible to interpret the interactions between the fillers and the polymeric matrix. Generally, a low value of the adhesion factor is an indication of good adhesion (or high degree of interaction between the two phases) due to the reduction of the macromolecular mobility induced by the presence of the filler [58].

### 2.3. Essential Work of Fracture (EWF) Approach

In order to characterize the polymers fracture, the linear elastic fracture mechanics (LEFM) approach is generally adopted. However, the characterization of fracture toughness by LEFM theory is difficult when relatively ductile polymers are considered due to the formation of a large plastic zone prior to crack initiation that violates the validity of LEFM approach. The fracture event of a relatively ductile polymer, having a marked plastic deformation zone at the crack tip, can be investigated by the essential work of fracture approach (EWF) originally suggested by Broberg [59] and then developed by Cotterell, Mai and co-workers [48,60,61,62]. According to this methodology, the fracture process zone is divided into two regions: an inner region (where the fracture process occurs) and an outer region (where the plastic deformation occurs). The total work of fracture follows this regions division and it can be separated in two contributions: the first contribution is related to the work spent in the inner fracture zone (work of fracture) and the work spent in the plastic deformation zone (non-essential work of fracture) [48].

For the application of the EWF theory, two different kinds of specimen can be used: the single edge notched (SENT) specimen and the double edge notched (DENT) specimen. Generally, the EWF approach is applied to thin sheets having a thickness between 1 mm and 0.2 mm [63]; the thickness of the specimen used (1.5 mm), although slightly higher than the standard range, is very low and consequently an attempt of applying the EWF approach was maintained.

**Figure 1:**
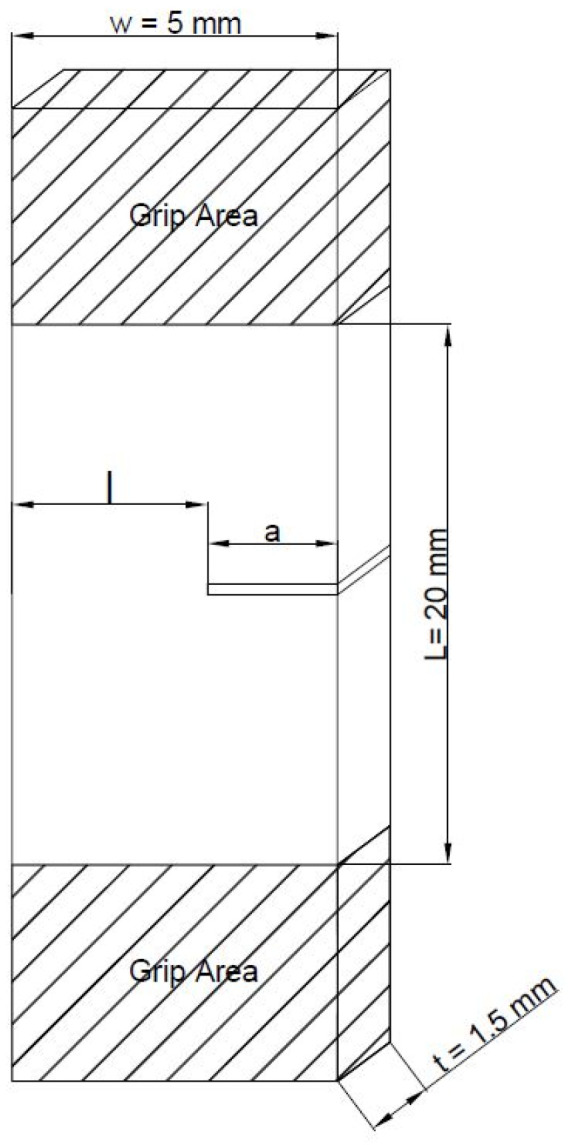
Schematic diagrams showing the single-edge-notched-tension (SENT).

The SENT specimen configuration illustrated in Figure 1 was adopted.

The total work of fracture, *W_f_*, is defined as:

(9) $$W_{f}=W_{e}+W_{p}\;$$

where *W_e_* is the work spent for the formation of two new fracture surfaces and it is spent during the fracture process and corresponds to the resistance to crack initiation. For specimens having a given thickness, *W_e_* is proportional to the ligament length, *l* (where *l* = *W* − *a*). In the Equation (9), *W_p_* is a volume energy, it is proportional to *l*^2^, and corresponds to the energy for activating the plastic deformation mechanism antagonists to the crack propagation. Consequently, Equation (9) can be rewritten in the following form (Equation (10)):

(10) $$W_{f}=w_{e}tl+\beta w_{p}tl^{2}\;$$

The specific total work of fracture can be written in the following form:

(11) $$w_{f}=\left(\frac{W_{f}}{tl}\right)=w_{e}+\beta w_{p}l\;$$

where *w_e_* and *w_p_* are the specific essential work of fracture and the specific non-essential work of fracture, respectively; *β* is the plastic zone shape factor while *t*, and *l* are related to the specimen geometry and correspond to the thickness and ligament length of the SENT specimen, respectively.

With the assumption that *w_e_* is a material constant and *w_p_* and *β* are independent from the ligament length, it is possible to plot Equation (11) as a straight line in a graph *w_f_* vs. *l*. Consequently, *w_e_* can be determined from the intercept of the *Y*-axis of the *w_f_* versus *l* plot, while *βw_p_* is the slope of the straight line. It must be pointed out that β depends on the specimen geometry and on the initial crack length so the straight line relationship can be obtained only if the geometric similarity is retained for all ligaments lengths.

## 3. Materials and Methods

### 3.1. Materials

The materials used in this work are listed below:

- Poly(lactic acid) (PLA), trade name Luminy LX175, provided by Total Corbion PLA (Rayong, Thailand). It is a biodegradable PLA derived from natural resources that appears as white pellets. This extrusion grade PLA can be used alone or mixed with other polymers/additives to produce blends/composites on conventional equipment for film extrusion, thermoforming or fiber spinning (D-lactic acid unit content about 4%, density: 1.24 g/cm^3^; melt flow index (*MFI*) (210 °C/2.16 kg): 6 g/10 min).
- Poly(butylene succinate-*co*-adipate) (PBSA), trade name BioPBS FD92PM, was purchased from Mitsubishi Chemical Corporation (Tokyo, Japan) and it is a copolymer of succinic acid, adipic acid and butandiol. It is a soft and flexible semicrystalline polyester that appears as matt white pellets (density:1.24 g/cm^3^; *MFI* (190 °C, 2.16 kg): 4 g/10 min).
- Triacetin (TA), or glycerol triacetate, was purchased from Sigma-Aldrich (St. Louis, MO, USA). It appears as an oily transparent and odorous liquid and it was used as biobased and biodegradable food contact plasticizer (CAS number: 102-76-1; density:1.16 g/cm^3^; boiling point: 258 °C; *Mw* = 218.20 g/mol).
- Wheat bran (WB) was provided by WeAreBio organic food; it appears as light brown powder and was used as filler for the PLA/PBSA plasticized blends formulations (CAS number: 130498-22-5; apparent density: 0.51 g/cm^3^; dietetic soluble fibre: 0.93% (p/p); dietetic insoluble fibre: 19.70% (p/p)).

### 3.2. SFT-IR Characterization

Infrared spectrum of bran was recorded in the 550–4000 cm^−1^ range with a Nicolet 380 Thermo Corporation Fourier Transform Infrared (FTIR) Spectrometer (Thermo Fisher Scientific, Waltham, MA, USA) equipped with Smart Itx ATR (Attenuated Total Reflection) accessory with a diamond plate, collecting 256 scans at 2 cm^−1^ resolutions.

### 3.3. SEM Analysis

Wheat bran morphology was investigated by scanning electron microscopy (SEM) with a FEI Quanta 450 FEG instrument (Thermo Fisher Scientific, Waltham, MA, USA). The bran powder was prior sputtered with a layer of platinum and then observed by SEM. The metallic layer makes the surface electrically conductive, allowing the electrons to generate the images. From the SEM images obtained, the dimensional distribution of the wheat bran powder was also evaluated. More than 200 particles were examined by using ImageJ^®^ software (National Institutes of Health and the Laboratory for Optical and Computational Instrumentation, Madison, WI, USA).

In addition, the composites morphologies were investigated by SEM, on a cryogenic fractured cross-sections of tensile specimens, to evaluate the fiber-matrix adhesion and the fibers dispersion inside the matrix.

### 3.4. Samples Preparation

PLA/PBSA blends were prepared using an Haake Minilab II (Thermo Scientific Haake GmbH, Karlsruhe, Germany) co-rotating conical twin-screw mini-extruder. Before processing the materials were dried in an air circulated oven at 60 °C for 1 day. For each extrusion cycle about 6 g of material, manually mixed with the additives, were fed into the mini extruder at 190 °C and 110 rpm. Subsequently the molten material was transferred, through a preheated cylinder, to a Thermo Scientific Haake MiniJet Mini-Injection Molding System for preparation of tensile dog-bone specimens (Haake bar type 3 with gauge dimensions: 25 × 5 × 1.5 mm^3^). The cylinder temperature was set equal to the extrusion temperature (190 °C) while the mold temperature was set at 50 °C. In order to completely fill the mold, 300 bar of pressure for 15 sec was set followed by a post pressure of 200 bar for 4 sec.

The blends compositions are listed in Table 1. On the basis of a previous work [18] a PLA/PBSA matrix containing 60 wt.% of PLA and 40 wt.% of PBSA was selected. The matrix was plasticized with Triacetin maintaining fixed the ratio between the matrix and the plasticizer (equal to 9), for all the formulations. The ratio between the matrix and plasticizer was set on the basis of literature work [40,41,42,64] in order to ensure a good processability and a high flexibility to counterbalance the effect of fiber addition that tend to embrittle the matrix.

### 3.5. Mechanical Characterization

Tensile tests were carried out, at room temperature, on Haake type 3 dog-bone specimens by an MTS Criterion testing machine model 43 (MTS System Corporation, Eden Praire, MN, USA) equipped with a load cell of 10 kN. The instrument is interfaced to a computer running MTS Elite Software in order to record the results of the tensile tests. The gauge separation was set at 25 mm and the crosshead speed was 10 mm/min. The main mechanical properties were collected. Tests were conducted after 3 days from the samples injection moulding and during this time the specimens were stored in a dry keeper (SANPLATEC Corp., Osaka, Japan) in controlled atmosphere (room temperature and 50% humidity). At least 10 specimens were tested for each blend composition and the average values of the main mechanical properties were reported.

For the EWF characterization the SENT geometry was used. The SENT specimens were obtained from the injection molded Haake type 3 specimens appropriately cut (size: 20 × 5 × 1.5 mm^3^). SENT specimens with different crack length (from 1.5 mm up to 3.5 mm) were produced by sharp notch and during the cutting process, compressed air was used in order to avoid the “notch closing” phenomenon caused by excessive overheating generated by the cutter. The ligament length was checked by optical microscope. The tensile load to the SENT specimen was applied using the previous mentioned MTS Criterion testing machine at a crosshead speed of 0.5 mm/min. At least five samples for each ligament lengths were tested and the average values of the area under the load displacement curves were reported.

Dynamic mechanical thermal analysis (DMTA) was carried out using a Gabo Eplexor^®^ DMTA (Gabo Qualimeter, Ahiden, Germany) with a 100 N load cell. At least three specimens were tested for each composition. The test bars (size: 20 × 5 × 1.5 mm^3^) were obtained by cutting the tensile specimens’ dog-bone specimens. The samples bars were mounted on the machine in tensile configuration. The temperature range adopted for the test varied from −80 to 120 °C with heating rate of 2 °C/min and at a constant frequency of 1 Hz. The relaxation temperature, associated with the glass transition, was taken at the maximum of the peak of the damping factor (*tanδ*).

## 4. Results and Discussion

### 4.1. Wheat Bran Characterization

**Figure 2:**
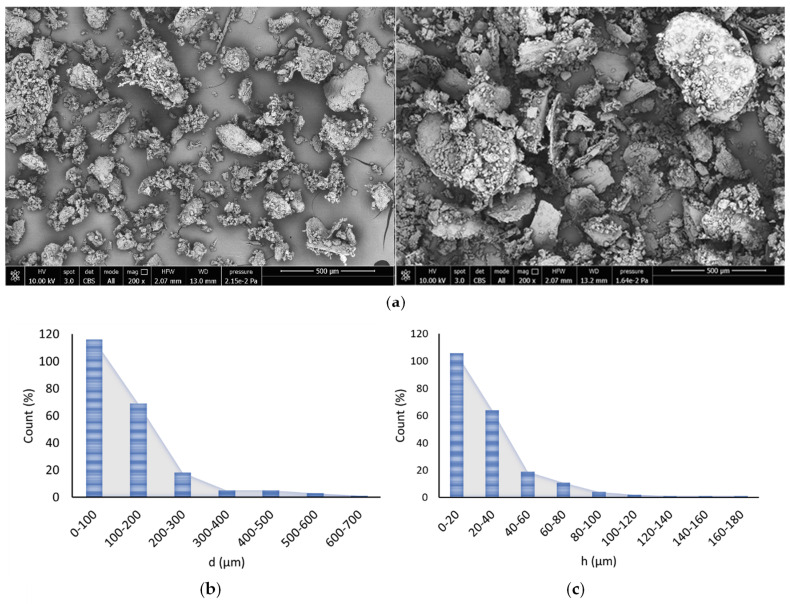
(**a**) SEM images at 200× of wheat bran fibers (**b**) average diameter distribution and (**c**) average thickness distribution.

The micrographs of wheat bran fibers (Figure 2a) show that bran particles have various dimension. Mainly the bran powder consists of platelets having low aspect ratio that forms agglomerates having an average size around 250–500 micrometers. However smaller size fraction in large quantity can also be observed. In the SEM micrographs the wheat bran fibers showed a low aspect ratio and their morphology is similar to flakes.

Consequently, for the fibers aspect ratio distribution a flake geometry was assumed where the filler aspect ratio, *a_r_*, can be defined as the ratio between the average fiber diameter (*d*) (calculated on the larger platelets surface) and the mean thickness of the platelets (*h*) according to Equation (12) [54]

(12) $$a_{r},platelets=\frac{d}{h}\;$$

In Figure 2b-c a sharp peak can be observed in the range of 0–200 μm and 0–40 μm for the mean fiber diameter and fiber thickness distributions, respectively. Thanks to the fibers distribution the mean weighted fibers lengths for the calculation of the mean fibers aspect ratio was carried out. In particular, a mean weighted fiber diameter of 219.76 μm and a mean weighted fiber thickness of 51.40 μm were obtained that correspond to a mean fiber aspect ratio of 4.27. Consequently, the composites obtained belong to the family of very short fiber composites [27].

**Figure 3:**
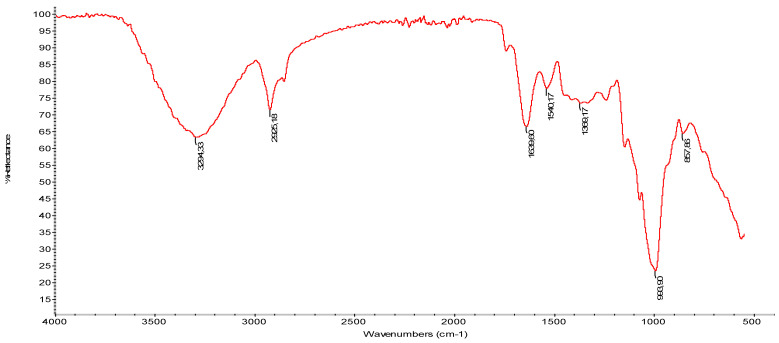
ATR spectrum of wheat bran fibers.

In the infrared spectra (reported in Figure 3) the hydrophilicity of the wheat fibers is highlighted by the very sharp –OH groups stretching in the region of 3650–3000 cm^−1^. The peak at 3294 cm^−1^ corresponding to –OH stretching was attributed to specific intramolecular hydrogen bonds of cellulose II [65,66]. The infrared spectra displayed a small adsorption band at 2925 cm^−1^, typical of the stretching vibrations of the C–H bonds in hemicelluloses and cellulose. The bands at 1639 cm^−1^ and 1540 cm^−1^, attributable to amide I and amide II vibrations, can be ascribed at the presence of the protein fraction in bran. Moreover, the shoulder at 1639 cm^−1^ is attributable to the acetyl and uronic ester groups of hemicelluloses or to the ester linkage of carboxylic group of the ferulic and p-coumaric acids of lignin [67]. Adsorption bands can also be observed near 1639 cm^−1^ and they are attributed to deformation of the C=O groups of xylan [68] that is the main component of hemicellulose.

### 4.2. Composites Results

**Figure 4:**
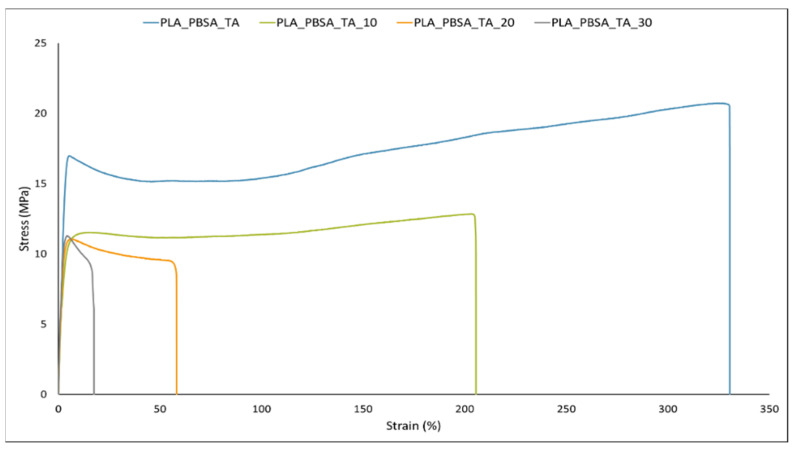
Stress-strain representative curves for plasticized PLA/PBSA composites.

Tensile tests results reported in Table 2 and Figure 4 evidenced that by increasing the bran content, a decrease in yield stress, stress at break and elongation at break was observed. As it can be expected, the bran addition caused a matrix embrittlement. This behavior is in line with literature papers on similar biocomposites [37,69,70]. The Triacetin addition was revealed a valuable approach to contrast the embrittlement caused by the bran fibers addition. At this purpose, it can be observed that up to 20 wt.% of bran content the elongation at break is still high (about 50%); also with 30 wt.% of Bran, despite the sharp drop down of elongation at break due to the high bran amount, the final elongation at break is still higher (17.3%) if compared to pure PLA (about 4% [6]).

The decrease in the stress at break with bran content is ascribable to a low adhesion between the matrix and the fibers that causes an ineffective transmission of the load from the matrix to the fibers.

**Figure 5:**
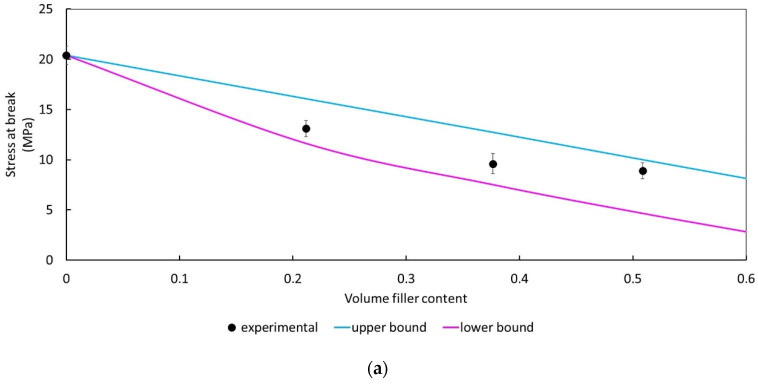

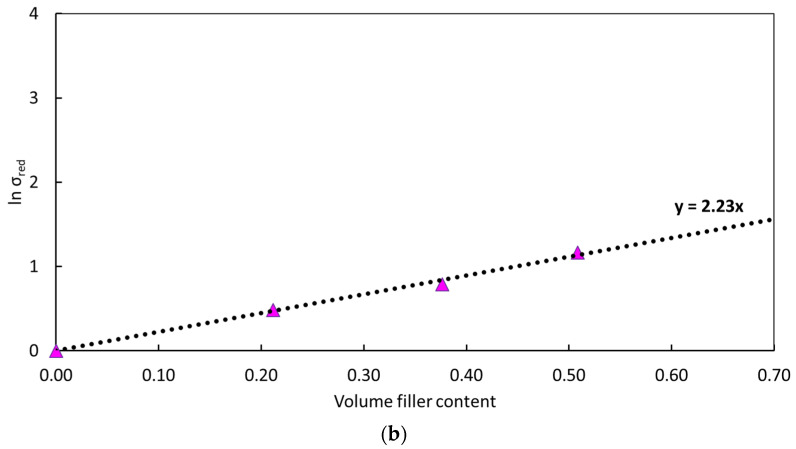
(**a**) Comparison between the experimental composites strength and the values predicted according to the analytical models illustrated in Section 2.1. Volumetric fractions are derived from the weight fractions calculated by using the density of each component taken from data sheets; (**b**) Pukánszky’s plot for PLA/PBSA plasticized composites.

The application of the analytical models for stress at break (Figure 5a) confirms that low adhesion exists between the wheat bran and the polymeric matrix. The experimental data lies exactly between the lower and upper bound confirming the good applicability of the analytical models to the analyzed system.

The poor adhesion is also confirmed by the B value obtained from the Pukánszky’s plot (Figure 5b) where a *B* value equals to 2.23 was obtained. Being the B value obtained greater than 2.04, the Equation (5) was used for the *IFSS* estimation and the value, reported in Table 3, was also compared to other similar composites systems containing natural fibers. The IFSS value obtained can be retained acceptable because it is inferior to its maximum theoretical threshold value achievable calculated by the Von Mises relationship [71] (*τ* = *σ_m_*/√3) equal to 11.78 MPa; furthermore the IFSS value obtained is comparable to similar bio composites described in literature.

**Figure 6:**
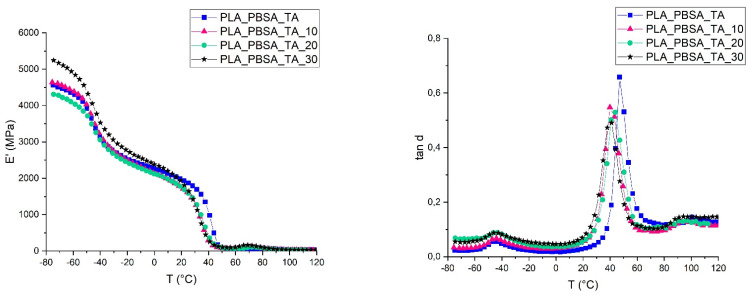
Variation of the storage moduli (E’) (**left side**) and damping factors (tan δ) (**right side**) with temperature for PLA/PBSA plasticized bran composites.

The addition of bran fibers causes a decrement of the storage modulus (Figure 6) that is similar to the decrement of the elastic modulus observable in Table 2. This decrement can be ascribed to the slight PLA chain scission induced by the bran addition that, as all natural fibers is sensitive to moisture, and caused the slight elastic modulus decrement and the shift, towards a lower temperature, of the PLA glass transition temperature [75,76]. Independently from the bran content, a shift of the PLA *tanδ* peak (corresponding to the PLA glass transition temperature) of about 5 °C is observed; while for PBSA no deviation in its *T_g_* (registered at around −44 °C) has been detected.

The height of the tanδ peak is similar for all the composites compositions. However, this height is lower if compared to the *tanδ* height of the matrix. This decrement is due to the introduction of the bran fibers that reduces the molecular mobility of the polymeric matrix [77]. This reduction of the molecular mobility is strictly correlated to the fiber-matrix adhesion: a good adhesion in fact is responsible of a major molecular mobility reduction. The adhesion factor (reported in Table 4) increases with the bran content indicating that the adhesion worsens increasing the fibers content. This result is in accordance to what was found in literature for other similar systems where a decrement of the interfacial shear stress with the increase of the volume fiber content was registered [27,78,79]. The difference in the high hydrophilic nature of the bran and the mainly hydrophobic nature of the matrix can be the main reason of the observed poor adhesion.

**Figure 7:**
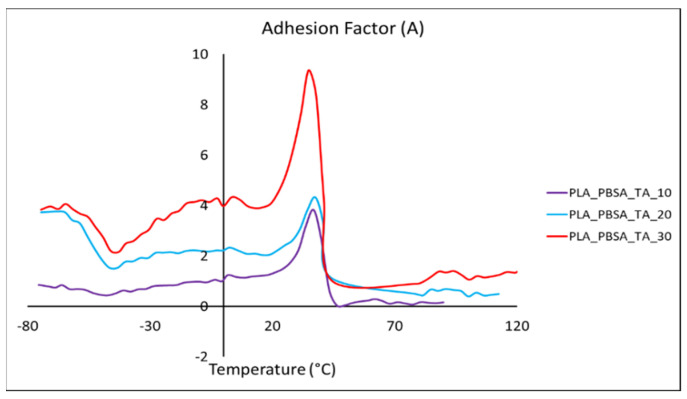
Adhesion factor vs. temperature for plasticized PLA/PBSA bran composites.

It is also worthy to notice, from the adhesion factor versus temperature illustrated in Figure 7, that the adhesion factor seems to be very sensitive to *T_g_*. Throughout the temperature range analyzed, the adhesion factor increases with the bran amount. This trend is a further confirmation that, at 30 wt.% of bran content, a net decrement of the mechanical properties of the composite occurs due to the slows down of the fiber-matrix interfacial adhesion. Moreover, the adhesion factor in correspondence of the PLA glass transition temperature suddenly increases reaching its maximum value. A similar behavior can be found in literature for other composite systems [57,80] and is ascribed to the higher mobility of the polymeric chains. Above the glass transition the difference in A value between the composites with different bran content are less evident than below the glass transition. Another increment of the adhesion factor, observable by another slight peak, is registered in correspondence of the PLA cold crystallization temperature (at about 90–110 °C [81]) for which the reorganization of the PLA polymeric chains caused another change in polymeric chains mobility, that is decreased due to the occurrence of crystals formation.

The influence of the presence of the wheat bran filler on the fracture of the material was also investigated with the aim of integrating it with the studies of interactions and adhesion.

**Figure 8:**
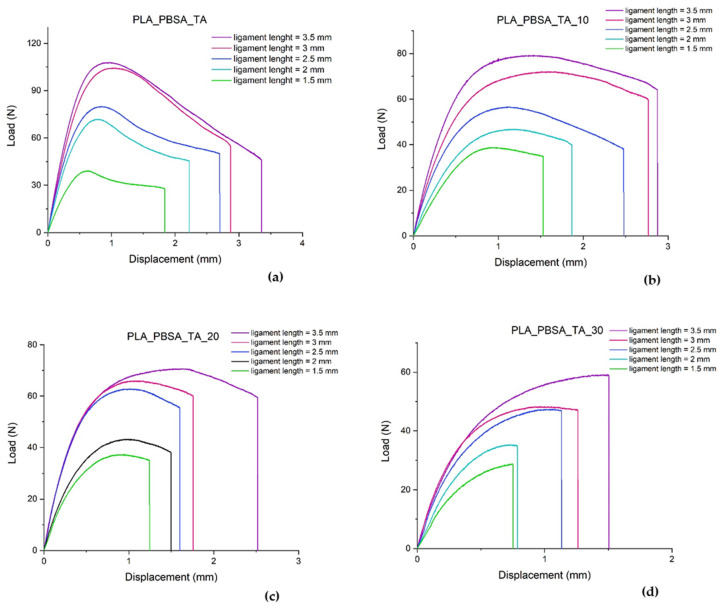
Load displacement curves of plasticized PLA/PBSA blend with and without bran at different ligament lengths.

The load displacement curves Figure 8a–d show a common behavior in the range of the ligament lengths examined (from 1.5 to 3.5 mm). As ligament lengths decreases, the maximum load decreases. Once the maximum has been reached, the materials undergo to a drop down of the load. By increasing the bran content, the load dropping is more marked and smaller displacement values were recorded. The fracture occurs suddenly after that the maximum load is reached and the introduction of the bran particles decreased the material ductility in accordance to the tensile test results.

**Figure 9:**
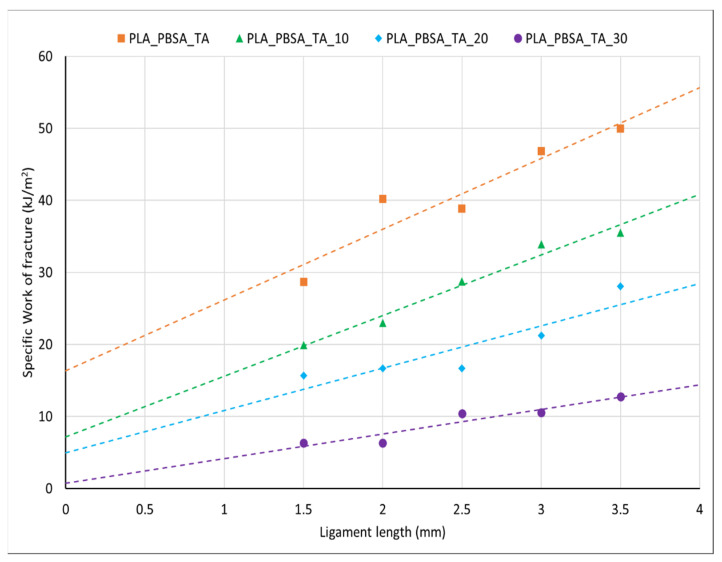
Specific total work of fracture w_f_ versus ligament length l for plasticized PLA/PBSA blend with and without bran.

The plot of the specific total work of fracture versus ligament length for the different blend compositions is shown in Figure 9 while the results of the EWF parameters are reported in Table 5. A good linear relationship between *w_f_* and *l* can be observed from the regression coefficients that lies in the range of 0.81 and 0.97 allowing the use of the calculated *w_e_* and *βw_p_* parameters. Comparing the data of fracture parameters emerges that both the essential work of fracture and non-essential work of fracture decreases with the bran amount. Since *w_e_* involves both the plastic deformation process of the necking ligament section and the work needed for the cracks to start growing [82], the maximum *w_e_* value will be reach for the material having the highest yield stress. This trend is confirmed, in fact increasing the bran content, a decrease of the yield stress was registered at which corresponds also a decrease of the essential work of fracture. The *βw_p_* increases with the material ductility and it decreases with the yield stress similarly to what was found by Arkhireyaya et al. [83] and in accordance to tensile test results. The lowest value of the essential work of fracture is registered for PLA_PBSA_TA_30 composite where the poor adhesion and the probable presence of agglomerates lowers abruptly the fracture energy.

The bran particles act as a stress intensification factor, reducing the cross section of the material and leading to a decrease of the tensile properties and fracture resistance. Reasonably because of the high dimension and low aspect ratio, the mechanism of pull—out (that generally leads to a toughness improvement even in the case of natural fibers reinforcement [84]) is not beneficially impacting the fracture resistance of these composites. Similar results were observed by Anuar et al. for composites with Kenaf fibers [85]. Thus, data about essential work of fracture are not largely available in literature, a systematic comparison with different natural fibers is currently difficult.

**Figure 10:**
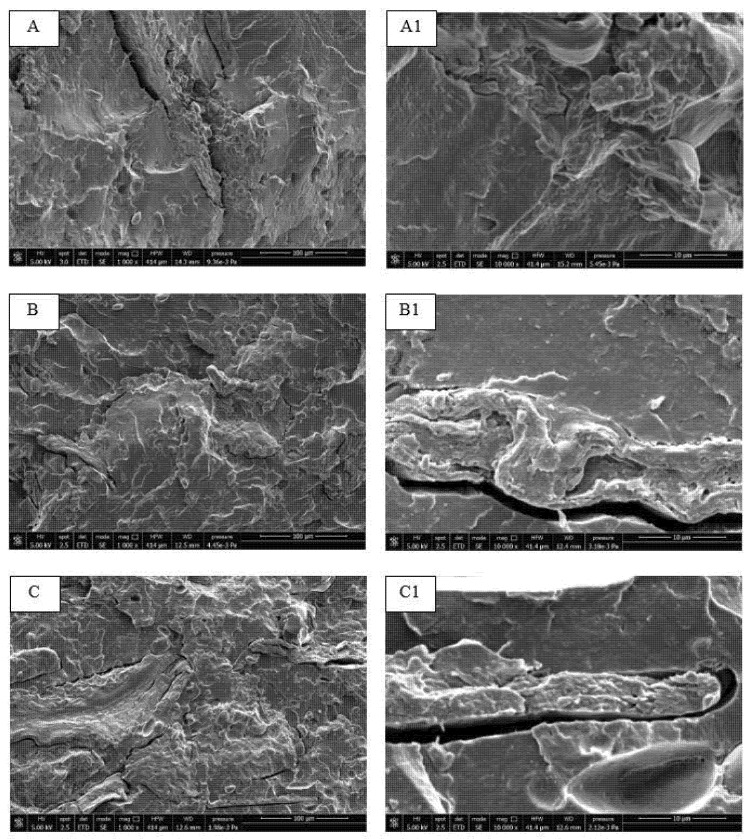
SEM micrographs of cryogenic fractured cross-section for: (**A**,**A1**) PLA_PBSA_TA_10; (**B**,**B1**) PLA_PBSA_TA_20; (**C**,**C1**) PLA_PBSA_TA_30.

SEM images (Figure 10) confirm the results. At 30 wt.% of bran (Figure 10C) the detachment from the matrix around the bran fibers becomes more marked. There is some fiber/matrix adhesion (although is not optimal) as evidenced by the magnification at 10 000X, for the composites containing 10 wt.% of bran (Figure 10A1). At 20 wt.% of bran the adhesion starts to worsening (Figure 10B1) the detachment of the bran fiber from the matrix, is present only in some regions of the fibers contours while in another there is continuity between the fiber contour and the matrix. At 30 wt.% of bran (Figure 10C1) an almost complete fiber detachment can be observed. Increasing the fiber content also increases the likelihood of finding fibers agglomerates that contribute to the decrement of the stress at break and to the essential work of fracture.

## 5. Conclusions

In order to extend the biobased polymers application and at the same time to reduce the final costs of the material, the design of suitable biocomposites containing natural fibers coming from industrial and/or agricultural waste is a feasible solution. In this work the addition of wheat bran fibers (from 10 to 30 wt.%) was investigated. In order to limit the excessive embrittlement caused by the bran addition a plasticized ductile matrix was chosen (PLA/PBSA matrix containing 60 wt.% of PLA and 40 wt.% plasticized with Triacetin). The evaluation of the interfacial adhesion (interfacial shear stress, *IFSS*) between the fiber and the matrix was carried out adopting analytical models based on static and dynamic tests. For the first time the essential work of fracture (EWF) approach was adopted on this type of biocomposites. The bran addition caused a decrement of the mechanical properties in parallel with the EWF reduction. In particular, the essential work of fracture is highly influenced by the presence and content of bran and this is ascribable to the high irregularity of filler geometry and presence of agglomerates especially where the content of filler is high. The combination of reduced adhesion between matrix- filler and increased intensification of stresses is responsible of the observed mechanical behavior as a function of the bran content.

## Figures and Tables

**Table 1:** Blends name and compositions.

Blend Name	Matrix (PLA 60 wt.%-PBSA 40 wt.%)	Triacetin wt.%	Wheat Bran wt.%
PLA_PBSA_TA	90	10	-
PLA_PBSA_TA_10	81	9	10
PLA_PBSA_TA_20	72	8	20
PLA_PBSA_TA_30	63	7	30

**Table 2:** Tensile data of PLA/PBSA plasticized blends.

Blend Name	Yield Stress (MPa)	Stress at Break (MPa)	Elongation at Break (%)	Young’s Modulus (GPa)
PLA_PBSA_TA	15.7 ± 1.8	20.4 ± 0.9	325.0 ± 19.9	1.9 ± 0.1
PLA_PBSA_TA_10	12.9 ± 0.8	13.1 ± 0.8	229.0 ± 32.6	1.61 ± 0.1
PLA_PBSA_TA_20	11.1 ± 1.3	9.6 ± 1.0	57.7 ± 10.8	1.6 ± 0.1
PLA_PBSA_TA_30	10.9 ± 1.0	8.9 ± 0.8	17.3 ± 2.7	1.6 ± 0.1

**Table 3:** IFSS obtained for the composites system analyzed compared with literature data of similar systems.

Reference	Matrix	Natural Fibres	IFSS (MPa)
Experimental	PLA/PBSA blend	Wheat bran	4.84
Aliotta et al. [6]	PLA	Cellulose	8.20
Li et al. [72]	PP	Hemp	5.84
Lopez et al. [73]	PP	Softwood fibers	3.85
Nam et al. [74]	PLA	Coir	4.56

**Table 4:** Bran composites adhesion factor calculated according Equation (8) at room temperature (25 °C).

Blend Name	A
PLA_PBSA_TA_10	1.5
PLA_PBSA_TA_20	2.4
PLA_PBSA_TA_30	5.2

**Table 5:** EWF fracture parameters for PLA/PBSA blends with and without bran.

Blend Name	w_e_ (kJ/m^2^)	βwp (MJ/m^3^)	R^2^
PLA_PBSA_TA	16.35	9.82	0.89
PLA_PBSA_TA_10	7.15	8.42	0.97
PLA_PBSA_TA_20	4.97	5.87	0.81
PLA_PBSA_TA_30	0.74	3.42	0.89

## Data Availability

The data presented in this study are available upon request from the corresponding author.
